# Perspective: sustainability challenges, opportunities and solutions for long-term ecosystem observations

**DOI:** 10.1098/rstb.2022.0192

**Published:** 2023-07-17

**Authors:** Akira S. Mori, Kureha F. Suzuki, Masakazu Hori, Taku Kadoya, Kotaro Okano, Aya Uraguchi, Hiroyuki Muraoka, Tamotsu Sato, Hideaki Shibata, Yukari Suzuki-Ohno, Keisuke Koba, Mariko Toda, Shin-ichi Nakano, Michio Kondoh, Kaoru Kitajima, Masahiro Nakamura

**Affiliations:** ^1^ Research Center for Advanced Science and Technology, The University of Tokyo, Komaba 4-6-1, Meguro, Tokyo 153-8904, Japan; ^2^ Graduate School of Environment and Information Sciences, Yokohama National University, 79-7 Tokiwadai, Hodogaya, Yokohama, Kanagawa 240-8501, Japan; ^3^ Japan Fisheries Research and Education Agency, 6F Technowave100, 1-1-25 Shin-urashima, Kanagawa-ku, Yokohama, Kanagawa 221-8529, Japan; ^4^ National Institute for Environmental Studies, 16-2, Onogawa, Tsukuba, Ibaraki 305-8506, Japan; ^5^ Conservation International Japan, 1-17 Yotsuya, Shinjuku, Tokyo 160-0014, Japan; ^6^ River Basin Research Center, Gifu University, 1-1 Yanagido, Gifu City 501-1193, Japan; ^7^ International Strategy Division, Forestry and Forest Products Research Institute (FFPRI), 1 Matsunosato, Tsukuba, Ibaraki 305-8687, Japan; ^8^ Field Science Center for Northern Biosphere, Hokkaido University, N9 W9, Kita-ku, Sapporo, Hokkaido 060-0809, Japan; ^9^ Graduate School of Life Sciences, Tohoku University, 6-3 Aoba, Aramaki-aza, Aoba-ku, Sendai, Miyagi 980-8578, Japan; ^10^ Center for Ecological Research, Kyoto University, Hirano 2-509-3, Otsu, Shiga 520-2113, Japan; ^11^ Kokusai Kogyo Co., Ltd. Shinjuku Front Tower, 21-1, Kita-Shinjuku 2-chome, Shinjukuku, Tokyo 169-0074, Japan; ^12^ Graduate School of Agriculture, Kyoto University, Kitashirakawa Oiwake-cho, Sakyo-ku, Kyoto 606-8502, Japan; ^13^ Tomakomai Experimental Forest, Field Science Center for Northern Biosphere, Hokkaido University, Takaoka, Tomakomai, Hokkaido 053-0035, Japan

**Keywords:** biodiversity loss, climate change, ecosystem services, equity

## Abstract

As interest in natural capital grows and society increasingly recognizes the value of biodiversity, we must discuss how ecosystem observations to detect changes in biodiversity can be sustained through collaboration across regions and sectors. However, there are many barriers to establishing and sustaining large-scale, fine-resolution ecosystem observations. First, comprehensive monitoring data on both biodiversity and possible anthropogenic factors are lacking. Second, some *in situ* ecosystem observations cannot be systematically established and maintained across locations. Third, equitable solutions across sectors and countries are needed to build a global network. Here, by examining individual cases and emerging frameworks, mainly from (but not limited to) Japan, we illustrate how ecological science relies on long-term data and how neglecting basic monitoring of our home planet further reduces our chances of overcoming the environmental crisis. We also discuss emerging techniques and opportunities, such as environmental DNA and citizen science as well as using the existing and forgotten sites of monitoring, that can help overcome some of the difficulties in establishing and sustaining ecosystem observations at a large scale with fine resolution. Overall, this paper presents a call to action for joint monitoring of biodiversity and anthropogenic factors, the systematic establishment and maintenance of *in situ* observations, and equitable solutions across sectors and countries to build a global network, beyond cultures, languages, and economic status. We hope that our proposed framework and the examples from Japan can serve as a starting point for further discussions and collaborations among stakeholders across multiple sectors of society. It is time to take the next step in detecting changes in socio-ecological systems, and if monitoring and observation can be made more equitable and feasible, they will play an even more important role in ensuring global sustainability for future generations.

This article is part of the theme issue ‘Detecting and attributing the causes of biodiversity change: needs, gaps and solutions’.

## 1. Introduction

The twin issues of climate change and biodiversity loss require integrative actions at regional and global scales, but policy discussions to address biodiversity loss lag behind those that directly address climate change [1]. With increasing concern over undesirable changes in ecological systems, the international community of biologists and ecologists has organized the Intergovernmental Science-Policy Platform on Biodiversity and Ecosystem Services (IPBES), following the model of the Intergovernmental Panel on Climate Change. IPBES released its first Global Assessment in 2019 [2]. While the socio-economic consequences of climate change, i.e. how anthropogenic climate change can have serious societal consequences, has been widely recognized for some time [3,4], society at large is only recently recognizing how human-driven changes in the biosphere can adversely affect all life on Earth [5]. Long-term observations are critically important to understanding biodiversity loss and climate change, but their usefulness will be determined by the effectiveness of collaborations between stakeholders across multiple sectors of society, including researchers in many disciplines and individuals involved in policy, business and funding bodies.

**Figure 1.**
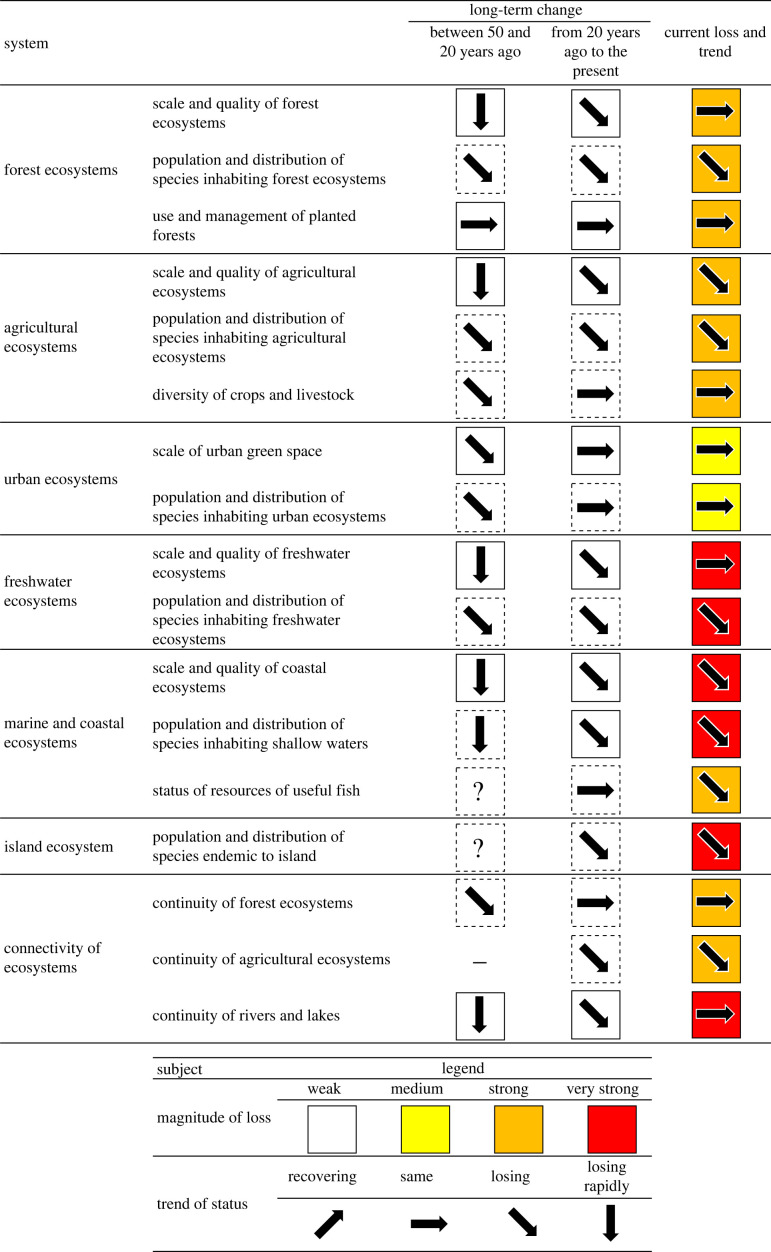
Biodiversity status and trends in Japan. Reprinted from Japan Biodiversity Outlook 3 (www.biodic.go.jp/biodiversity/activity/policy/jbo3/generaloutline/index.html). Permitted by the Ministry of the Environment, Japan.

**Figure 2.**
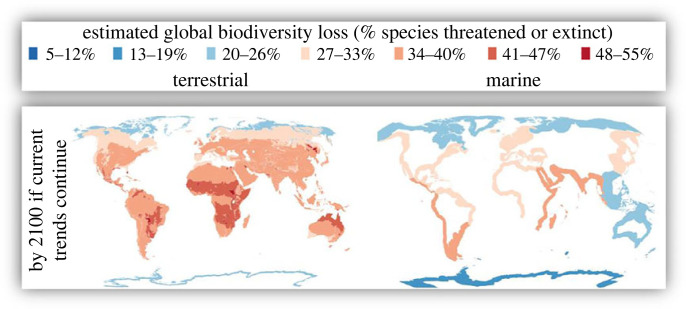
Expert estimates of changes in global biodiversity in terrestrial biomes (left column) and marine realms (right column) by 2100 if current trends continue. Reprinted and with permission from Isbell *et al.* [11]. Maps are based on the Mercator projection.

With the growing concern about the trend and status of natural capital [5–7], especially the increasing social recognition of the value of biodiversity [8], urgent actions are needed to detect changes in biodiversity [9,10]. The opinions of experts in a wide range of disciplines, from the natural sciences to the social sciences, often play key roles in informing policy at the national (figure 1) and global scales (figure 2) [11]. Despite differences in these and many other assessment frames, experts are consistent in their opinions (figures 1 and 2) and are calling the current situation a crisis [11]. However, these alarming changes cannot be attributed to key drivers and accurate projections cannot be made, without robust estimations of how current trends deviate from the baseline [12–16]. Ecosystem monitoring, especially long-term [16,17], is critical to detecting trends and changes. Note that Gonzalez *et al.* [16] proposed that causal attribution should come before, because causal models should guide observations and monitoring design. All these steps (i.e. causal modelling, observation, estimation, detection and attribution) are important to support policies that have a critical role and responsibility in ensuring the functioning of the ecosystem and the diverse, long-term benefits and intergenerational well-being it provides [16].

At the regional scale, long-term monitoring has been instrumental in identifying critical drivers of ecosystem changes [18–22]. For instance, in central Japan, long-term monitoring revealed that species interactions were a prominent driver of fish population dynamics [18]. This was a key contribution to the theoretical understanding of ecosystem stability—a long-standing study theme that is important in theory and practice [23–28]. However, conducting ecosystem monitoring at similar spatio-temporal resolution across the globe in a coordinated manner remains a challenge. Nevertheless, this example highlights the critical importance of long-term monitoring and the potential benefits it can bring to understanding and managing ecosystems. Emerging techniques and opportunities, which can be combined, like environmental DNA (eDNA) and citizen science [29–32], offer promising avenues for detecting changes in biodiversity and their impact on ecosystem services (e.g. fisheries [30]). However, effective monitoring methods can be costly, and monitoring sites are often located in remote areas or regions that face difficulty in ensuring long-term funding to support ecosystem observations. There are still many barriers to establishing and sustaining ecosystem observations at a large scale with fine resolution. Addressing these barriers would create new opportunities for advancing our understanding of ecosystem changes.

**Figure 3.**
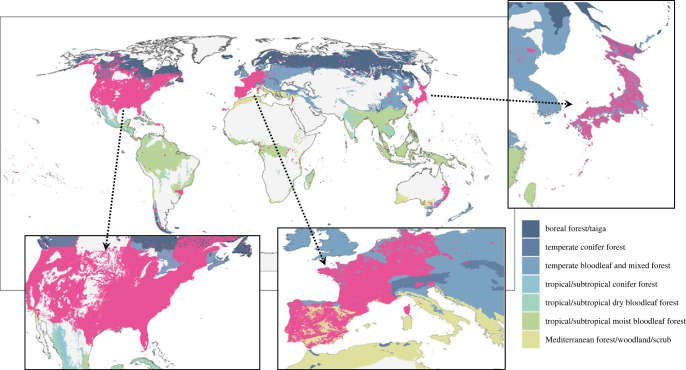
Location map of forest biodiversity monitoring sites: the magenta dots are the monitoring sites of the Global Forest Biodiversity Initiative (https://ag.purdue.edu/facai/data/gfbi.html) [34,35]. Different forest biomes [36] are indicated by different background colours. Some regions with abundant monitoring sites are highlighted. Note that the plot locations in New Zealand are not publicly available and are therefore excluded from this visualization: data are abundant in the country (https://www.gfbinitiative.org/metadata-gfb1). Maps are based on the Robinson projection.

First, it is important to fill data gaps, which include geographical and taxonomic biases in monitoring changes in ecological and socio-ecological systems. For example, biodiversity inventories are typically more comprehensive near locations that offer convenient access, infrastructure and logistics [33]. These gaps make joint monitoring of biodiversity and anthropogenic factors challenging (see also figure 3 for regional biases in forest biodiversity monitoring sites worldwide [34,35]). Second, extensive infrastructure is often required for observations that cannot be systematically established and maintained in many locations [37]. Finally, it is difficult to establish a global systematic network of monitoring across sectors and countries with different economic and cultural status, requiring an equitable solution [37]. These barriers are interconnected and emphasize the need for joint monitoring of biodiversity and anthropogenic factors, the systematic establishment and maintenance of *in situ* observations, and equitable solutions across sectors and countries to build a global network.

Some argue that global targets should be worded in an inclusive manner to accommodate unique national circumstances to ensure that each target is relevant to and obligatory for each party, i.e. minimum national targets [38]. While we see the benefits of this system [38], achieving these global targets would require globally standardized efforts in assessing planetary health [39,40]. This is metaphorically equivalent to a medical check-up: for a comprehensive assessment of an individual's health condition, no organs or body parts can be ignored. This approach therefore needs a feasible way to be inclusive across different stakeholders. To illustrate the challenges, opportunities and solutions of ‘global' sustainability in ecosystem monitoring, it is important to shed light on regions that are not necessarily major players for cultural, linguistic or other reasons [41–45]. Therefore, to provide suggestions for the continuing policy discussions on international cooperation, we focus on specific examples and emerging frameworks, primarily in Japan.

Note that Japan has a long tradition of ecosystem monitoring [46–49], but the data, efforts, emerging opportunities and associated outcomes are not fully visible and accessible to the global stakeholders, as is often the case in non-English speaking countries [41–43]. We thus believe that highlighting Japan's ecosystem monitoring efforts is valuable in drawing attention to the importance of comprehensive and systematic monitoring programmes for understanding ecosystem changes, and also highlights the need for making such information accessible to the global community. In other words, focusing on less- and under-representing regions is crucial when considering globally concerted efforts [44]. This is important to emphasize the need to address societal reasons to address challenges in ecosystem monitoring, in addition to ecological reasons. With these issues in mind, we describe how ecosystem monitoring can be implemented in a feasible and equitable manner; this is important for ensuring the sustainability of the planet for future generations.

## 2. Expanding monitoring data and schemes: gaps

Recognition of the importance of long-term ecological research (LTER) has expanded in recent years; LTER studies provide new insights into the factors and processes underlying ecological change and improve our understanding of how ecosystems are responding to environmental changes. The International LTER (ILTER) network is a global long-term, site-based research network that seeks to build ecological and socio-ecological knowledge [20,50,51] using long-term data on ecosystem structure and function [47,52]. According to the Group on Earth Observations (GEO; https://www.earthobservations.org), the concept of biodiversity observations encompasses satellite imaging, remote sensing, and ground-based data, to monitor the status of and assess changes in natural environments in order to provide policy support. The international network of long-term ecological monitoring and research has been expanded, but there are still regional biases indicating the need for filling spatial and taxonomic data gaps (e.g. https://www.ilter.network/network/global-coverage).

Here is a brief summary of history of the efforts to monitor natural systems. One of the early global collective efforts is the International Geosphere–Biosphere Programme (IGBP), which was launched in 1987 to coordinate global and regional international research on the interaction of Earth's biological, chemical and physical processes. Many of the IGBP projects have now transitioned to the Future Earth, with greater focus on the social and economic aspects of the human system in shaping Earth systems (see also [53]). One of its global research projects is bioDISCOVERY (https://futureearth.org/networks/global-research-networks/biodiscovery/), which is aimed at fostering international interdisciplinary research activities and policy support based on observations, from remote sensing to *in situ* measuring of biodiversity. The bioDISCOVERY aims to add value to existing research by focusing on building scientific networks and coordinating research by connecting researchers and integrating methodologies. This is consistent with our discussion of equitable solutions (described later).

Now, such initiatives are further increasing, which are multi-scaled so as to respond to policy and other needs under the changing environment. The GEO Biodiversity Observation Network (GEO BON; https://geobon.org) is organized to improve the acquisition, coordination, and delivery of remote sensing and *in situ* biodiversity observations and related services to decision makers, the scientific community, and other stakeholders. To address the need for regional observations of biodiversity, Asia-Pacific BON was launched in 2009, engaging scientists and institutions through regional activities in the Asia-Pacific region [54,55]. In 2022, the Europa Biodiversity Observation Network (EuropaBON; https://europabon.org) was officially endorsed as a regional BON. These efforts have strengthened the ecosystem observation network spatially. For the LTER networks, there are many national-level schemes, such as US-LTER in the United States (https://lternet.edu/) and JaLTER in Japan (http://www.jalter.org/). As described previously [21], advancing ecological theory requires network-level integration, and there is an emerging opportunity to integrate big data to test ecological and socioecological hypotheses with greater spatial and temporal relevance [56–58]. However, as noted, the spatio-temporal distribution of data remains uneven. Below we provide some specific examples to illustrate the issues.

### (a) Data gaps in terrestrial realms

Among terrestrial biomes, there is increasing recognition of the critical importance of ensuring the functionality of forest ecosystems for climate solutions and beyond [1,56,59,60]. However, even now, humanity has a surprisingly weak understanding of how ecological processes operating in forests respond to climate change: there are complex, unresolved questions about how climate change will affect forest health, how it will affect the performance of forests as carbon sinks, and how it will alter the ecosystem services they provide [61–63]. Continuous and long-term observations of the current state of and changes in ecosystems, as well as of human activities such as land-use change, are needed to obtain comprehensive data for the model. To give a specific example, examining the Global Forest Biodiversity Initiative map (https://www.gfbinitiative.org) reveals substantial biases in the distribution of long-term monitoring of tree communities (figure 3 [34]). In some countries where forest inventories are monitored at fine spatial resolution, monitoring data are so rich that the coordinates of the observation points can be used to draw a map of the whole country (e.g. Japan and the USA). That is, there are few gaps between observation points in these areas (also see the electronic supplementary material, figure S1). On the other hand, very limited data are available for many heavily forested countries (e.g. in the global South), illustrating that data are unevenly distributed across the global forest biome (figure 3), which presents a major obstacle to realization of biodiversity benefits, including nature-based solutions for climate change [56,64,65].

Soil biodiversity provides another example of the biased spatial distribution of long-term monitoring in terrestrial biomes. Specifically, belowground biodiversity such as invertebrates, fungi, bacteria, and protists in soils and freshwater sediments play critical roles in supporting numerous ecosystem functions and services, from climate stabilization to food production [66–68]. In the context of the Global Biodiversity Framework of the Convention on Biological Diversity, assessing the status and trends of soil biodiversity information would provide a direct assessment of the extent to which countries are meeting post-2020 biodiversity targets [39]. Notwithstanding their importance, data on belowground life are much sparser than those for aboveground life (Global Soil Biodiversity Initiative; https://www.globalsoilbiodiversity.org), [69] and thus vertical biases exist even within regions with rich aboveground vegetation data. In addition to these vertical (within an ecosystem) and horizontal (across regions/countries) biases in belowground biodiversity data, the temporal dynamics are largely unknown, as most available data are based on snapshot monitoring. This may change, as Soil BON, part of GEO BON, emphasizes the need for longer-term monitoring of soil biodiversity data and for collecting concrete data on temporally and spatially explicit soil biodiversity and ecosystem function indicators [39].

### (b) Data gaps in marine realms

The global distributions of marine species are shifting in response to climate change [70], resulting in changes of local ecosystem services through changes in local biodiversity and ecosystem functioning [71]. Understanding the impacts of climate change on fisheries and aquaculture via changes in local biodiversity has been one of the most important topics addressed by international organizations [72–74]. Various attempts are underway to use observation data to create adaptation and mitigation measures to address the problem of declining global fish stocks. Adaptive management is viewed as an experiment in resource management strategies, from which managers iteratively test hypotheses and then adapt their policies accordingly (often referred as *learning by doing*) [75]. Significant uncertainty exists in the management of fish stocks and aquaculture in a changing marine environment. Lack of data on interactions between fish species and environmental factors in fishery grounds makes it difficult to capture signals of temporal changes in important components of the system, such as fluctuations in fish stocks. That is why systematic monitoring is necessary. Monitoring is also important to sustain seaweed communities. For instance, coastal areas of Japan are now intensively monitored (electronic supplementary material, figure S1), which helped understand how the distribution of the foundation species in coastal communities throughout Japan has changed over decades owing to climate change and the impacts of the Great East Japan Earthquake and the associated tsunami [49].

In the search for adaptation measures, global fish redistribution is being monitored by using data from scientific bottom trawl surveys [72]. Assessment of the status and accessibility of the data revealed that more than 55% of the metadata collected were not publicly available, suggesting that data availability is the most important obstacle to assessing species redistribution under global climate change. To improve data availability, an open database of eDNA data is being constructed in Japan (box 1*a*, All Nippon eDNA Monitoring Network (ANEMONE); https://db.anemone.bio). Similarly, analyses of eDNA and oceanographic observation data enable us to understand and to predict changes in the distribution patterns and life histories of various fish species in response to climate change, which is essential for developing new adaptive measures for local fisheries and aquaculture.

Box 1. **Rapidly accumulating citizen science data. Here, we provide some newly developed, ongoing initiatives driven by citizens. Citizens play a central role in biodiversity monitoring and data archiving:**

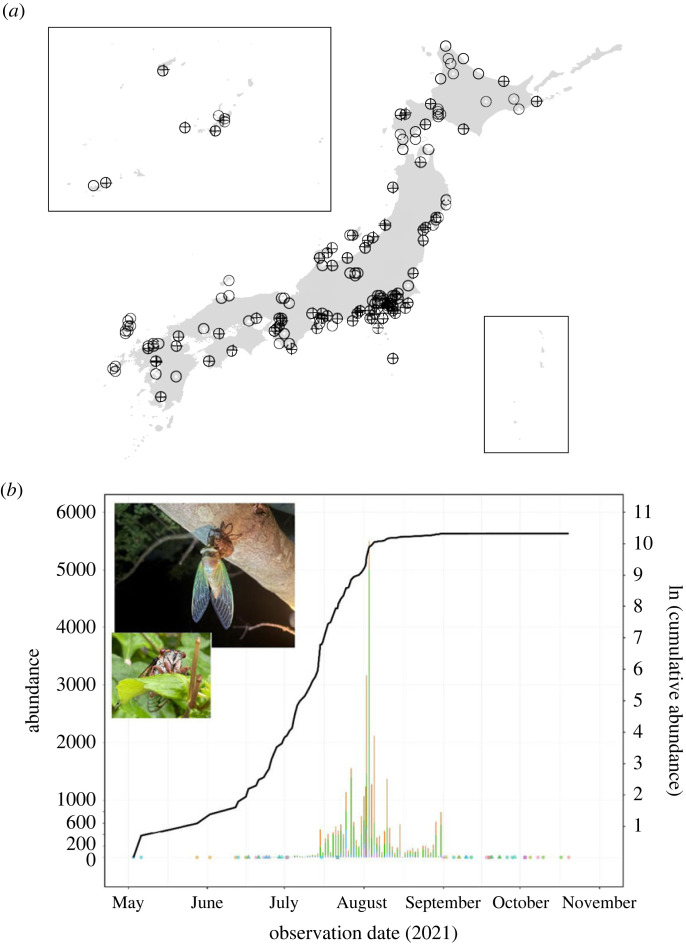

(*a*) Sampling points of eDNA under the initiative of All Nippon eDNA Monitoring Network (ANEMONE). Open circles indicate locations where observations are being made by research institutions and scientists. Crosses indicate locations where observations are being conducted by citizens (Earthwatch Japan; www.earthwatch.jp/). This initiative has a open database (ANEMONE DB; https://db.anemone.bio). The map was downloaded from the Digital National Land Information from the Ministry of Land, Infrastructure, Transport and Tourism, Japan (same as figure 2).

(*b*) This graph is based on a call by the Japan Broadcasting Corporation (NHK) for observations of different cicada species throughout Japan. Shortly after this new citizen science programme began, the number of observations exponentially increased. Such citizen science is expected to play an important role in furthering our understanding of species whose life histories and distributional status are unknown. As part of this programme, a citizen reported sighting a species of cicada that was thought to be locally extinct. Bars with different colours represent observations of different species. Note that abundances of 1, 2 and 3 are represented by circles, triangles, and squares, respectively, because they cannot be distinguished if shown as bars. Different coloured bars indicate observations of different species. As an example, two of the many photos provided and archived by citizen scientist are also shown (credit: NHK, Japan).

Lastly, there are vertical biases in data collection in the water, similar to those found in terrestrial realms. For example, while coastal shores are receiving attention in the context of ocean-based climate interventions and mitigations (as described later), the consequences of these interventions on deep-sea ecosystems are largely unknown, highlighting the need for data to support evidence-based policy-making [76].

### (c) Biodiversity to support ecosystem functioning and services

Note that, despite the expansion of international networks and initiatives aimed at monitoring biodiversity and ecosystem functioning, data gaps remain a significant obstacle to understanding feedbacks between these system components [77–79]. These gaps exist not only in terms of observations of organisms, but also in understanding their contributions to ecosystem functioning and services. Efforts such as identifying the essential ecosystem service variables, based on the experience of the essential biodiversity variables exist [80], but further data collection, integration and sharing are necessary to link biodiversity information with ecosystem services and nature's contributions to people. Addressing these data gaps is crucial for detecting changes in socio-ecological systems, projecting their possible consequences, and making available knowledge more relevant to policy in order to achieve sustainability in the real world.

Across different biomes and realms, biodiversity and its relationships to ecosystem functioning and human well-being depend on feedbacks within and between these system components [56,77]. Thus, it is necessary to focus on linkages and in particular on feedbacks in order to use observation data to help detect changes in socio-ecological systems and project their possible consequences. However, making available knowledge more relevant to policy, which will help achieve sustainability in the real world [78,81,82], still faces many difficulties. Because feedback between human and ecological subsystems is an important area for guiding interdisciplinary research and discourse [77], considering feedback processes can increase the effectiveness of investments in ecosystem observations.

## 3. Ensuring meaningful long-term monitoring: challenges

### (a) Funding and coordination constraints

Individual site efforts and developing networks between them are undoubtedly important to gain better insights about how ecosystems will respond to ongoing environmental changes, especially if implemented in a globally coordinated manner (e.g. [20,51,83–86]). In addition to this, identifying key, ubiquitous mechanisms that can be widely relevant to different systems sometimes requires costly ecosystem manipulations. Establishing long-term monitoring is not easy, especially for systems that require heavy expenditure and infrastructure, because long-term funding is very limited. For instance, CO_2_ flux monitoring is playing an increasingly important role in forest science, especially given the growing need to capture carbon dynamics at the biosphere–atmosphere interface [87]. However, even for passive measurements, large-scale flux monitoring systems using canopy towers, for example, are not easy to install and maintain. Also, when more active approaches are required, such as free air CO_2_ enrichment [88] or artificial warming treatments [89] in forests, there are even greater difficulties in implementation and maintenance. Even for field-based data collection, which costs a pittance by comparison, it is a struggle to ensure financial stability [90]. In Japan, the Japan Meteorological Agency dramatically reduced its level of long-term phenological monitoring in 2021, probably owing to the difficulty of securing funding [91]. This is unfortunate since some observations have lasted for hundreds of years (e.g. the blooming phenology of cherry trees has been observed for over 1200 years (since 812 AD; [92])). Thereby, systematically establishing *in situ* monitoring sites, and especially maintaining them in a long run, are the real challenges.

More to the point, there needs to be a better relationship between university research and long-term monitoring. Long-term observational data would provide the basis for science and education broadly, not just ecology (as emphasized in the US-LTER website: https://lternet.edu). However, many early-career researchers who need near-term outcomes in their career development avoid studying themes that require long-term observations for acquisition of new data. It may be more realistic to focus on research themes for which long-term observations have already been conducted. However, long-term observations are often suspended or discontinued for financial or social reasons, resulting in lost opportunities for the next generation of researchers. There are thus many issues to be discussed, such as providing long-term funding stability for universities that contribute to monitoring and designing monitoring schemes that are attractive to career researchers and that can be analysed at an early stage (e.g. combining a space-for-time substitution approach with a real-time series). Despite some successes, such as the German Biodiversity Exploratories [93–95], the long-term funding stability of observational research to drive both science and education remains a major challenge. Although the next generation of researchers will be responsible for carrying on ecosystem observations, the value of long-term observational data as an asset to be passed on to the next generation has not been duly appreciated.

Another issue to be discussed is that policy and economic reasons are driving investments that are less effective than they could be. Some ongoing schemes are somewhat redundant for assessing general trends in biodiversity and natural capital at the national scale. For example, nationwide ecosystem observations have been conducted under different schemes even within Japan (electronic supplementary material, figure S1). While much of the data are publicly available and contribute to scientific endeavours and policy development [46,96–98], these monitoring programmes are not effectively coordinated across different sectors. This is partly because they are led by different governmental organizations and have different specific purposes such as risk assessment of species extinction (e.g. [99]), biodiversity monitoring (e.g. [46,96]), land and catchment management (e.g. [97]), and pest control. Specifically, monitoring programmes led by the Ministry of Environment are mainly aimed at detecting climate change impacts on biodiversity, but changes in carbon stocks are observed at the same time. Monitoring led by the Forestry Agency, on the other hand, began for accurate identification of forest resources (the National Forest Inventory), but in recent years data have been used to improve the accuracy of forest carbon sink calculation. Therefore, better allocation of limited resources and budgets is an issue that should be discussed beyond science.

### (b) Societal needs

There has been a sharp rise in interest in climate change, leading to the rapid expansion of carbon monitoring efforts in recent years. Disclosure frameworks such as the Taskforce on Climate-related Financial Disclosures (https://www.fsb-tcfd.org) have made companies realize that climate change mitigation, by reducing their own emissions and beyond, are top business priorities. This, combined with a growing recognition of the importance of nature-based solutions for climate change [1,65,100–102], has led to an increase in the business sector's interest and the inflow of funds through carbon crediting schemes. For instance, salt marshes, mangrove forests, seagrass beds and macroalgal beds, are considered one of the five key ocean-based actions to achieve the limit of the Paris Agreement [103]. However, these blue carbon ecosystems are being seriously degraded owing to sea desertification and reduced genetic diversity in macroalgal and seagrass beds [104,105], making their protection and monitoring crucial [106]. Countries such as the USA and Australia have already included blue carbon ecosystems in their greenhouse gas inventories [107,108]. In Japan, efforts are underway to establish methods for calculating carbon sequestration in oceans [109]. These efforts aim not only to offset emissions but also to recognize the co-benefits of ecosystems, such as coastal protection, fisheries enhancement, and biodiversity enrichment. Similar efforts to increase multiple benefits also exist in terrestrial ecosystems, especially in forests. However, owing to difficulty in quantification (e.g. [110]) and possible trade-offs between sectors (e.g. forest carbon versus agriculture; [111]), caution is necessary when considering carbon credit schemes.

Equivalent efforts to address the challenges of biodiversity change are emerging (e.g. the taskforce on nature-related financial disclosures; https://tnfd.global), but they are lagging behind those for climate change. Business sectors will need to understand their impact on biodiversity and take mitigation measures [5], but it is not yet clear how and what should be monitored to understand the impacts and changes [8]. Biodiversity offsetting has long been proposed, but it faces substantial technical and ethical difficulties [112]. Biodiversity is changing outside companies' business footprints owing to various anthropogenic factors. To ensure that such changes are not overlooked, a framework linking societies and ecosystems is needed, and generalized indicators would be essential for establishing linkages beyond a direct relationship between a member of society and a particular site [5,8]. A science-based design is necessary to know if an action taken for business reasons will yield true sustainability consequences. The current scheme does not account for actual socio-ecological consequences, such as whether an action taken in the course of economic activities ultimately benefited or harmed biodiversity and ecosystems. Currently, companies and other actors are required only to take actions, without measuring their actual impacts. To move forward, we need to establish a framework that scrutinises and publicises the ecological outcomes of decisions. This can only be assessed through long-term ecological observations, which are essential for achieving sustainability [113].

Currently, ecosystem impact assessments require only a minimal level of accuracy and precision, but discussions and negotiations are underway to improve these measures, especially through the Kunming-Montreal Global Biodiversity Framework (GBF) of the Convention on Biological Diversity [114], which envisions the 2030 targets and 2050 goals. The locate, evaluate, assess and prepare process of the Taskforce on Nature-related Financial Disclosures (TNFD; https://framework.tnfd.global/the-leap-nature-risk-assessment-process/) is also related to the GBF Targets, particularly, Target 15, which calls for various measures to encourage and enable businesses and financial institutions to regularly monitor, assess, and transparently disclose their risks, dependencies and impacts on biodiversity. Accurate monitoring is crucial for supporting nature-related risk and opportunity assessments. While quantifying the impacts of industrial activities on biodiversity and nature's benefits to society is challenging, now is the time to rethink our social structure and revise our approach to evaluating the real value of natural capital.

## 4. Sharing monitoring data, tools and facilities: opportunities and solutions

### (a) Investing in activities in space or on land

A variety of novel opportunities exist for gathering and sharing data. Remote sensing plays a key role in this regard. The most shared and well used example is Landsat data, which already has a long history of Earth observations and is available for free [115]. Laser scanning is increasingly becoming more feasible and less expensive to acquire three-dimensional data (examples of different airborne laser scanning methods are described in box 2). Importantly, when the IGBP was launched no one would have imagined that there would come a time when data from high-resolution laser scanning of forests and terrestrial topography from the International Space Station would be made publicly available for free (Global Ecosystem Dynamics Investigation (GEDI); https://gedi.umd.edu).

Box 2. **With the development of monitoring technologies such as laser imaging detection and ranging (lidar), it is becoming increasingly feasible to acquire three-dimensional scanning data.**

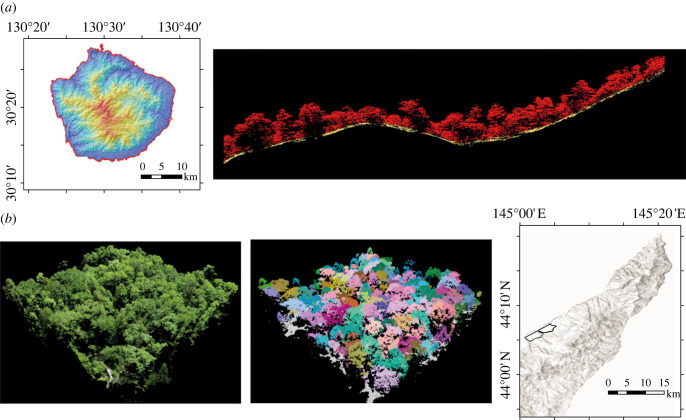

(*a*) Airborne laser sensing was conducted throughout Yakushima Island (approx. 504 km^2^), southwestern Japan. The data were acquired from an aeroplane (laser density = 10 points m^−2^). This helped to identify one of the largest trees in the nation. The right panel shows an example of three-dimensional forest structure along the valley-ridge transect.

(*b*) Three-dimensional forest structure in a forest restoration area (shown with black solid lines in the map in the right panel) in Shiretoko National Park. Data were acquired by drone (laser density greater than 600 points m^−2^). The left pane is an image of the sensing, and the middle panel is shown for individual trees (shown with different colours; see the electronic supplementary material, figure S2 for an animated image). Such fine-resolution data allow stakeholders to quantitatively assess the outcomes of decades of ecosystem restoration activities [116–119] (https://100m2.shiretoko.or.jp). While these technologies are not yet fully available all over the world, they are becoming more feasible in many areas, even in inaccessible locations, and thus are expected to facilitate monitoring of ecosystems.

Space-borne monitoring is playing a critical role in detecting changes in the climate and social and ecological systems and their interactions [120,121], and it is expected to play further key roles [122]. Despite its fundamental role and the need to invest in space exploration, there can be another way of thinking about the allocation of resources and funds. In many parts of the world, data on the spatial distribution of organisms (aboveground, belowground and in the water), especially their temporal dynamics, are still lacking. Although many approaches to connecting satellite data and *in situ* observations of biodiversity and ecosystem functions have been discussed, spatio-temporal and methodological gaps remain [122,123]. Despite huge advancements in *in situ* data collection, further investments are necessary to better integrate these different types of data [123]. It may be more efficient to invest part of available funds in filling these data gaps on the ground and to training local experts to ensure *in situ* biodiversity monitoring around the globe before making huge investments in space-based sensing. For accurate estimations of ecosystem changes from space and air, on-ground quantifications and model sophistication are crucial. Also, investments in human resources are vital, especially actions from the nations that have more fundings and technologies to facilitate those with limited capacities in terms of budgets, skills, and human resources. As such, difficulties still remain but, without the climate and biodiversity crises, GEDI would not have been developed and these valuable open data would not have been shared around the world. Hence, this and other next investments—which can be viewed as new opportunities arising from the crisis—must be worthwhile.

Another example comes to mind: the exploration of Mars. There is no doubt that Mars exploration, which is enormously expensive, makes sense as a basic scientific endeavour. However, Mars exploration assumes that at some point in the future, humans will migrate from Earth, or that at some point it might become necessary to terraform Mars for humanity. If these scenarios come to pass, long-term data on social systems and ecosystems will be critical to help us design and create ecosystems from scratch. We do not deny the importance of making huge investments for hundreds or thousands of years into the future. However, we argue that it is only within the next 10–30 years that we have the opportunity to ensure that we leave a beautiful environment on Earth for the current and next generations. In other words, if we do not solve these problems within the next few decades, our investments in the distant future will be wasted. To fulfil our responsibility to future generations, we need to focus on health inspection and care of our planet now.

### (b) Feasible options and equitable solutions

Changes in the global environment must be detected and attributed on meaningful time scales, but we must consider the possibility that a new global monitoring programme may not be ready to launch in time. Suppose, for example, that a new monitoring site is established in an unexplored tropical montane forest. It may take 20 years or more to obtain new information on the response of this site to climate change and its impact on the carbon cycle. Or, in the case of arctic tundra vegetation, where many processes such as plant growth and decomposition of organic matter are slower, it may take even longer to accurately determine how the changing climate affects regional carbon dynamics. A feasible option to overcome this difficulty is to rely on the space-for-time substitution approach, complementary with the time-series approach, which has already provided many critical insights (e.g. the PREDICTS database that compiles data from across a broad range of taxa [124–126]). Indeed, these different approaches could be combined: new monitoring could start with a larger number of sites covering a gradient of interest (e.g. climate or land use). Then a subset of the sites could be monitored permanently. Owing to the smaller number of sites, the effort would be reduced, but the time series generated would allow validation of the predictions based on the space-for-time substitution approach. Another approach is to revisit sites where a census was once made and to collect similar data to compile very-long-term monitoring data. This approach is not particularly new and has often been implemented in the past for plants and animals (e.g. [127,128]), but the efficiency and cost-effectiveness of expanding this approach globally has not yet been properly tested (see also [129]). Note that while this approach is often relatively inexpensive and easy to implement, it has downsides. First, it may not provide exhaustive coverage of all biomes, regions, and taxonomic groups, as it is limited by the historical bias of where censuses have already been conducted. Second, it is not a substitute for continuous monitoring, which can detect small changes in the health of Earth and early warning signals. Thus, although it is not ideal, it does present a valuable opportunity to acquire data on a time scale that we cannot afford to extend into the future.

Other non-standard ways to collect data should also be considered here. The first that comes to mind is that we should keep an eye on eDNA, as it is easy and inexpensive, and anyone can participate in monitoring [130,131]. Not so long ago, it was unimaginable that genomic data would be so readily accessible, another equivalent to the aforementioned example of space-based observations. Although some imperfections remain (i.e. noise can creep into eDNA data during extraction, polymerase chain reaction amplification, or library preparation; there are taxonomic biases here also), this technical advancement shows real promise for making ecosystem observations feasible. A number of new tools and methods have emerged to overcome technical challenges and thus eDNA is therefore expected to play a critical role in facilitating biodiversity observations. A major advantage of this tool is that if analysis and bioinformatics were performed at a hub research institution, this would allow for a seamless and open process of data verification and archiving (box 1*a*). Also, noteworthy example (again from Japan) is the nationwide collaborative research effort using eco-plates to assess the multifunctionality of soil microorganisms; high-resolution data (from 33 natural forest sites throughout Japan) are quickly and efficiently being obtained and made publicly available by hub research institutions [132]. This method also sheds new light on how ecosystem functionality, supported by biodiversity [77,81] such as in soil microbes [116], can efficiently be quantified. Of course, caution must be exercised. In particular, the Nagoya Protocol, a 2010 appendix to the Convention on Biological Diversity, has introduced extremely challenging regulatory changes in biodiversity sampling, especially in countries with high biodiversity [133]. Despite the practical challenges involved in transporting materials and specimens to hub facilities beyond legislative boundaries, these new tools provide an opportunity for collaboration between different stakeholders and can significantly increase the number of observation points across space and time without the enormous expense of satellite networks.

It is noteworthy that these observation tools are also highly compatible with citizen science [29,31,32]. The stakeholders, as mentioned above, include citizens as well as scientists. Continuous observation by citizens, performed in a non-burdensome and therefore sustainable way, can provide high-resolution monitoring data in both space–time and time–space dimensions (box 1). In fact, citizen monitoring of biodiversity has attracted attention in recent years, owing largely to the development of new survey methods; but surveys and sampling have been conducted not only by citizens, of course, but also by staff of nongovernmental organizations, museum curators, government officials, and academic researchers. Of course, collaboration among stakeholders is important, and there are many ways to facilitate these collaborative opportunities, using images, video and audio as well as eDNA. Biodiversity monitoring in citizen science has a long history, with programmes continuing for decades in some areas [134,135]. In such long-term monitoring projects, wildlife is often documented by observations and specimens, and the data may not have been digitized or made publicly available. Making data publicly available requires the creation of user-friendly interfaces and databases for ease of use by citizen scientists. Citizen science still poses some challenges for scientific research, but given the success of online citizen science networks such as eBird (https://ebird.org/home) and iNaturalist (https://www.inaturalist.org), there is great potential. Citizen science initially focused on animals (especially birds), but the focus has now been extended to other taxonomic groups. These new opportunities may compensate for or even improve upon the institutional models currently facing difficulty. Specifically, some citizen-based monitoring programmes have started (see box 1*b* for an example of insect monitoring) since the Japan Meteorological Agency substantially cut the long-term monitoring programmes (https://adaptation-platform.nies.go.jp/plan/institute_information/information_01.html)—this is another example of how a crisis can create an opportunity.

These tools and approaches would make it possible to implement a standardized protocol for continuous collection of data across countries, if a laboratory in any country became a hub. This approach could fulfil needs such as Soil BON's desire for coordinated, high-resolution monitoring on a global scale [39]. Ideally, it would be beneficial to collect raw data using a standardized protocol during field and other observations, which will facilitate health checks of the planet, prompt responses by people, and especially early warnings that are critical for the detection-attribution framework (also see [16]). However, in socio-economically unequal relationships, developed countries could provide funding, facilities and technical support to areas where fundings and skills are in short supply. Situations where developing countries become *pro bono* providers of samples for developed countries must be avoided. More specifically, while many databases are currently being created to collect and maintain biodiversity data in an integrated manner [124,136–139], there are commendable efforts to gather data that vary across different regions of the world, standardize them into a consistent format, and make them publicly available. It is important to note that currently, there may often be situations where developed countries operate existing integrated databases, and researchers from the global South and low-income countries merely contribute data. This user-donator pattern could result in a bias in terms of rewarding and crediting scientific research, which must be avoided based on the experience of the previous colonial structure. We must remember to ensure an equitable scheme for data acquisition, analysis, and its use across countries, as emphasized for many situations of international collaborations and collective actions [44,140,141]. International networks like ILTER and GEO BON should further assist in the ecosystem observations, in which data might be archived in different formats and in local languages, to help store metadata in a standardized format with representative languages like English and beyond, to ensure wider accessibility and equitable distribution of these benefits.

Filling knowledge gaps among scientists on how to use novel monitoring techniques should be prerequisite for the mutual, equitable collaboration needed to establish better ecosystem monitoring. Collaborations for ecological research activities among scientists from developed and developing countries via on-site research training are effective and should be established. For instance, the ILTER Nitrogen Initiative Training Course in 2016 welcomed scientists from all over the world to a hands-on training course in a field setting, performing sampling and data acquisition with both classic techniques (e.g. soil incubation) and cutting-edge technologies (e.g. laser N_2_O isotopomer analysis), as well as data syntheses, to boost the skills and knowledge of the attendants (see also [52]). Through such programmes, scientists from developing countries can receive training in not only the fundamental aspects of ecosystem monitoring but also the potential applications of novel monitoring techniques such as eDNA, multispectral remote sensing and continuous monitoring of water/gas chemistry by spectroscopy. Another example of the collective efforts is seen from the Global Wood Decomposition Network [86]: wood decomposition was quantified in 133 sites spanning six continents, using the standardized material and protocol, to tease apart the influence of decomposers (microbes and termites) and climate. For this experiment, standardized woodblocks were not available to participants in some countries owing to quarantine and trade regulations, budgetary limitations and other reasons, but the study materials were sent from others to them. While the study was designed to be less cost-demanding and less technical to facilitate concerted efforts globally, there was still a barrier but there was a solution. Likewise, collective efforts are essential to ensure truly meaningful ecosystem monitoring across both spatial and temporal dimensions [77]. Globally standardized efforts using the above tools and approaches in an inclusive manner would be optimal for assessing the health of our planet.

### (c) Networks across different sectors

In addition to the advancement of detection and attribution frameworks in ecological systems, machine learning and artificial intelligence are essential to realizing a sustainable future [142,143]. These tools have become indispensable in combining remote sensing and ground data, conducting spatio-temporal extrapolations, and projecting plausible futures. They are now widely used in business and industry, especially in the economic and financial sectors.

In areas with high biodiversity, industry can play an important role in collecting ecological data while also supporting local economies [5,144]. For example, we speculate that tourists could be trained to collect data on wildlife sightings, water quality, or other ecological indicators, which could then be used for research and monitoring purposes. Also, the industry often operates in remote areas where ecological monitoring is essential, suggesting that companies could provide access to funding and resources that could be used for scientific activities including ecosystem monitoring. Similarly, agriculture is one of the largest drivers of biodiversity loss [2] and often conflicts with forest-based measures for conserving biodiversity and carbon [111], but it is also essential for providing food and livelihoods. As exemplified in the TNFD, scientists could help develop sustainable industrial practices that support both biodiversity and human well-being.

Collaborating with the business sector for analysis of ecosystem observation data has proven highly effective [5]. Of course, the products of such collaborations require expert assessment by scientists, not artificial intelligence. In summary, cross-sector collaboration not only contributes to win-win solutions between sectors but also is essential for win-win solutions for both nature and society.

## 5. Way forward

As the value of biodiversity becomes increasingly recognized, sustained ecosystem observations are needed to detect changes in biodiversity through collaboration across regions and sectors. However, there are many barriers to establishing and sustaining large-scale, fine-resolution ecosystem observations. To effectively implement the detection and attribution framework, we call for collective actions to jointly monitor biodiversity and anthropogenic factors, systematically establish and maintain *in situ* observations, and promote equitable solutions across sectors and countries to build a global network. We hope that our proposed framework and examples can serve as a starting point for further discussions and collaborations among stakeholders across multiple sectors of society, to ensure global sustainability for future generations.

Although we primarily focused on specific examples from Japan to illustrate the sustainability challenges, opportunities and solutions for ecosystem monitoring, we believe that the content is common and helpful across countries and regions. Most of the initiatives and efforts taking place in many parts of the world, especially in non-English speaking countries, are not well recognized worldwide (also see [44,145]). Monitoring data, even when publicly available, are often in the local language rather than English, limiting their use beyond the country or language area. Local data collection efforts frequently go unnoticed outside their origin country unless reported through English-speaking media and outlets. Synthesizing studies published in non-English-language—from major languages such as Chinese and Spanish to local or indigenous languages—is key to overcoming the widespread lack of local, context-dependent evidence and facilitating evidence-based conservation globally [43]. The reported evidence serves as the foundation for strengthening the detection and attribution sequence, including the development of causal models and the reinforcement of the observational framework based on them [16]. However, we speculate that the underuse of vast amounts of information and evidence, owing to cultural and linguistic barriers [41–44,145,146], has led to significant unrecognized losses and opportunity costs in advancing the framework to address biodiversity issues. We hope that these insights on how to sustain ecosystem monitoring in a manner that is feasible and equitable can be expanded to solve global challenges in socio-ecological systems.

As has been emphasized recently [147], ecological science, which provides many benefits to society, relies on long-term data, and thus neglecting the basics of monitoring our home planet further reduces our chances of overcoming environmental change: we must remember that it is *not too late*. We have noted that crises can be opportunities, and that especially in difficult times, we must avoid wasting opportunities (see ‘the pity-to-waste-a-good-crisis idea’; [148]). In the climate and biodiversity emergency, society has already faced many challenges, including the postponement of summits owing to the COVID-19 pandemic since 2020. However, the global health crisis has allowed us to consider how working remotely, while difficult, can provide many opportunities for collaboration among people who live in different parts of the world. Now, the time is ripe to take the next step towards detecting changes in socio-ecological systems. Monitoring and observation, if implemented in a more equitable and feasible way, will play key roles in ensuring global sustainability for future generations.

## Data Availability

The data are provided in the electronic supplementary material [149].
